# Data and data illustrations supporting the analysis of transcripts from interviews exploring the views and experiences of young men and their parents/guardians regarding testicular health.

**DOI:** 10.1016/j.dib.2020.106106

**Published:** 2020-08-04

**Authors:** Caroline MacDonald, Maria Burton, Robert Carachi, Stuart O'Toole

**Affiliations:** a School of MVLS, University of Glasgow, University Avenue, Glasgow G12 8QQ, United Kingdom; b Royal Hospital for Children, 1345 Govan Rd, Glasgow G51 4TF, United Kingdom; c Faculty of Health and Wellbeing, Sheffield Hallam University, Sheffield S1 1WB, United Kingdom

**Keywords:** Testicular torsion, Adolescent health access, Adolescent health, Testicular health, Qualitative research

## Abstract

Evidence shows young men have poor outcomes from testicular torsion directly attributable to delay in presentation to hospital [1]. Only a third to a half of adolescents present within 6 h with testicular pain, [2,3] There is poor understanding of why adolescents delay in presenting with testicular pain. The authors started without an a-priori hypothesis and designed a thematic qualitative research protocol to explore the phenomena is a naturalistic setting [4,5] . Sixteen young men (11–19 years) and their parents or guardians underwent semi-structured interviews, directed by a topic guide which evolved with subsequent interview findings. Young men were recruited from out of school clubs to minimise the bias associated with schools or hospital recruitment, and were naïve to testicular disease. Verbatim transcriptions were coded, categories and themes formed and final concepts derived utilising a framework methodology. The figure included shows the initial topic guide. The data tables presented show the emergent themes and the final code book. The authors have utilised the analysis to explore the factors impeding young men in presenting early to hospital with testicular pain [6]. The authors feel the data tables and raw data will be of interest to other researchers interested in adolescent health, health access, public health, linguistics and healthcare qualitative methodology.

**Specifications Table**

**Table:** 

**Subject**	Perinatology, Pediatrics and Child Health
**Specific subject area**	The views and experiences of young men and their parents regarding testicular health
**Type of data**	Figure, Table
**How data were acquired**	Semi-structured interviews, recorded by electronic Dictaphone and transcribed verbatim. Inductive thematic analysis with one-sheet -of-paper, iterative reflexivity and framework methodology analysis. Informed consent and assent was obtained from parents and participants prior to interview.
**Data format**	Secondary and raw data. Raw data associated with Table 1 and 2 can be found at Mendeley Dataset: DOI: 10.17632/cvfxm2j3w8.2
**Parameters for data collection**	In order to explore the views and experiences of the normal young men regarding testicular health the authors purposefully did not recruit those who had torsion or other ailment of testis, or those with chronic disease. The target population was 11–19 year old young men, of mixed demographics. The authors chose not to recruit from schools as this is known to bias the responses. The young people were given £15 voucher as an incentive, and asked to choose a chaperone of their choice. All chose their parent or guardian. The initial protocol was for one young person with one or two chaperones, but two deviations off protocol occurred and gave depth to the data. As per standard qualitative methodology, the findings from data analysis and reflexive practice was feedback to altered and refine the topic guide as the interviews progressed. Recruitment and interviews continued until data saturation was achieved.
**Description of data collection**	Participants were recruited from out of hour clubs, such as football or weight loss clubs. Semi-structured interviews with the young men (aged 11–19 years) were undertaken at the club, hospital or home with their chosen chaperone present. All but one chose for the interviews to be undertaken at home. Consent for audio recording, transcription and publication was undertaken. The interviews followed a topic guide but allowed to run with breadth or depth of topic. The interviews were performed, recorded onto electronic Dictaphone and transcribed verbatim. They were then analysed using classic thematic techniques.
**Data source location**	City/Town/Region: Sheffield and Glasgow Country: England and Scotland Raw data held: Mendeley Dataset: DOI: 10.17632/cvfxm2j3w8.2
**Data accessibility**	Repository name: Mendeley Dataset Direct URL to data: http://dx.doi.org/10.17632/cvfxm2j3w8.2 No data access control issues, consent for publication obtained from participants.
**Related research article**	C M MacDonald, ‘*Why Adolescents Delay with Presentation to Hospital with Acute Testicular Pain: a qualitative study’,* Journal of pediatric Surgery, doi.org/10.1016/j.jpedsurg.2020.06.041

## 1 Value of the data

• These data are important because they are the first to describe adolescent males’ and their parents views and experiences of testicular health within naturalistic environments.
• Researchers interested in adolescent and family attitudes to health and access to health care will benefit from the data.
• Whilst analysed in the context of delay in presentation to hospital with testicular pain, the data could be utilised to investigate huge ranges of different issues. For instance: adolescent acute health access, parental-adolescent views of healthcare, e-literacy, family use of the internet for health information or family response to interviewing with a doctor.
• This data may be of value additionally to qualitative linguistic investigators and those investigating interview technique or the role of a doctor in healthcare qualitive research.

## 2 Data description

**Fig. 1:**
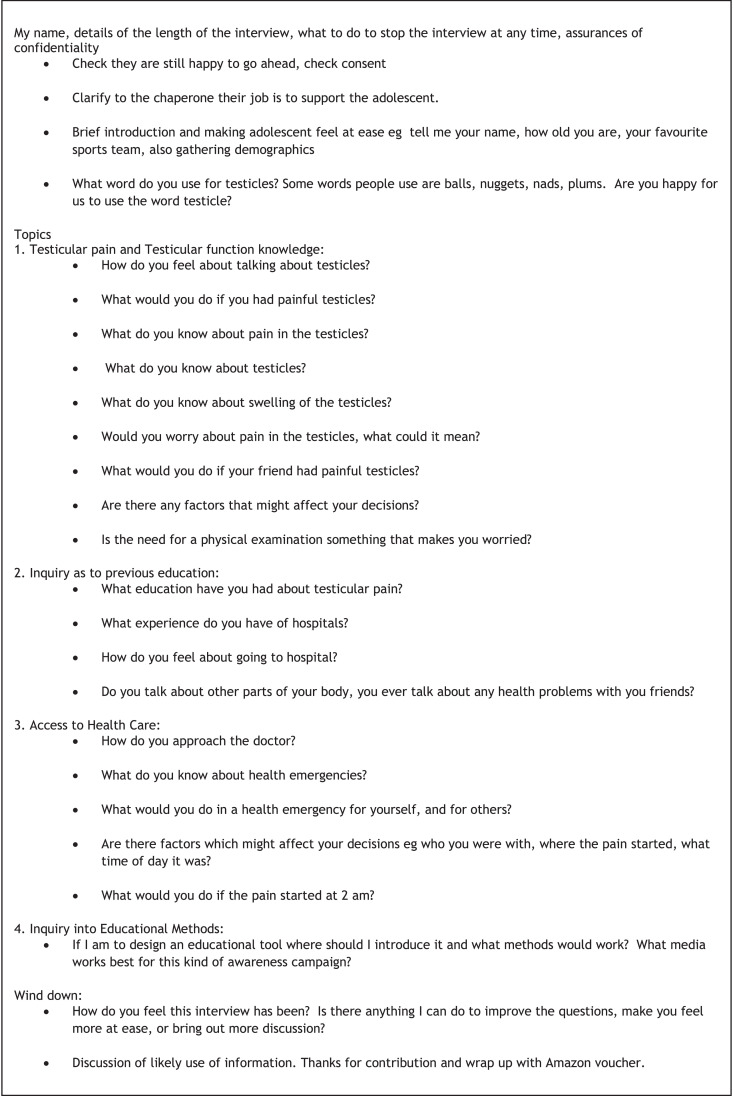
Initial topic guide and format for the semi-structured interview.

Fig. 1. describes the topic guide initially derived to explore the views and experiences of adolescents males regarding testicular pain. This underwent adaption with the iterative process.

Table 1. evidences the quotes within the transcript data which were utilised in developing the categories and themes within the associated manuscript, which focused on describing possible barriers to early presentation with testicular pain. This displays, in an auditable fashion, the closeness of the interpretation to the original data, and shows validity of theme development. Table 2 describes the main themes that were derived from the categories which came directly from the coded data. It gives granular detail of the topics revealed during the process of the interviews.

**Table 1:** Quotes supporting the main analytic themes; Key I: interviewer, P: participant, M: mother, F: father.

Theme	Representative Quotes
Reliance on parents for all aspects of healthcare	I: What do you think you would do if you had pain in your testicle? P:Tell Mum and Dad M: He's never been to the doctors by hiself anyway. I: Would you come to A&E on your own? P: I won't be able to get here. I: Would you ever feel comfortable calling an ambulance yourself? P: No
Young persons’ knowledge of testicular function and pathologies	I: have you heard about sperm? P:No. I: Have you heard of testosterone? P:No. I: if you had pain in your testicle would you know what to do? P:No I think if you asked everyone in my year. None of them would know! I: Have you ever heard of a twisted testicle or twisted ball? P: No. I:Did you know this could happen? P:No. I: What do you think would be the reason that young people maybe don't come to hospital in time with a twisted testicle? P: It's not knowing about it.’
Young Persons’ experience of health education and testicular health specifically	I: Tell me what you have learnt in your biology lessons. P: Just learnt about reproduction and what the parts do P: Well they made us label the parts and stuff and then they talked about sex education. I: So, do you know what testicles are for? P: No not really. M: So knowing the labels wouldn't increase or decrease the chance of you going to someone if you had pain? P: No.’
Young people's Views and Experiences of health issues	I: Do you think needing to have an operation would put you off? P: I won't really want to go under for something like that M: But you would compared to the alternative P: Yeh I'd much rather. You wouldn't look forward to the operation P: I think ummm people if they had [torsion] that they would think: ‘that's quite funny’ but if you have cancer I think people take [it] more seriously and wouldn't joke about it
Young Persons Health information seeking behaviour	I: If you had found a lump in your testicle what would you have done about it? P:Hmm told my dad. I: would ever have a look for information? P:Nah, just probably just talk to my friends. I: If you had anything you were worried about health wise would you ever turn to an internet resource. P: Probably not unless it's a definite NHS resource or definitely proven web site. P: Well, I know not to believe everything I see and not to just click on the first thing that comes up and, just like, if I've got a cough and I could search for a cough and it could come up with I'm gonna die tomorrow.
Ability for the Young Person to discuss male Issues with Parents	P: I'd probably tell yous [indicating Mum after being asked who he'd talk to about health problems]. M: The other day we were talking about some random stuff, it sudden comes up in conversation. Say something comes on telly we'll talk about that. M: Dad's a bit funny, he's kind of don't talk about it, but he's awkward whereas I talk about it, I try to be more open. […] It's a personality rather than a gender thing. M: Because a lot of kids are embarrassed to come to their parents. I wouldntnae be. I'm a parent I have to deal with things like that.
Psychological aspects of adolescent life affecting ability to discuss testicular health	P: I think some people might think it's embarrassing, […] but I don't, yeah. I: You might not say straightway. Why? P: In case I don't know, it might go away. I: how do you feel about talking about testicles or balls? P:Kind of like, I don't enjoy talking about it but I'm not, like, that bothered with–I don't really–I'm not super-embarrassed about it, I wouldn't say. I: is that because it was specifically the testicle? P: Not really it's kinda weird talking about your body I: would you be embarrassed if someone asked you about your sex life or something like that? P:No. I:If it's a doctor say? P: No.
Sociological aspects of adolescent life affecting ability to discuss testicular health: ‘Youth culture’	P: Cause I think your arm is always on show and everyone notices your arms but it's a bit different with your testicles. And I mean, reasonably, they're always hidden away. I: Would you be worried about people finding out at school? P: My friends would laugh and then take the mick and then, like, take it serious. I:How do you feel about you know if you are unwell how do you feel about telling someone like generally? P:I feel ok. I:It's not like you feel [you] have to be strong and tough. If you are unwell you feel that's ok to talk about it? P:Yes.
Parental Knowledge and Education	I: Have you ever heard about anyone having a health problem with their testicles? M: No I: if it twists it's an emergency and you have to get to hospital within 6 h otherwise you can lose your testicle M: I've never heard of that. [..]quite scary isn't it, quite serious M: With you saying that [CI describing torsion], it was a first date and a guy I kinda knew, and it got really sore and he went away and I saw him like 20 years later, and still didn't know what had happened. […] And now I am really surprised I didn't know about it.
Information Available for Parents or Guardians Regarding Testicular Torsion	I: do you think you'd know what to do? M: I think I would just call the doctor for advice, I'd dunnae really know what to do. M: I think for me in this day and age if something goes wrong in general health you just google. Google this and google that, we don't need to see a doctor we'll jus google it M: Sometime ‘googling’, but that can be bad as you think the worst [Father enters and reiterates ‘the worst’] so you can get very mixed information can't you? it can be helpful but sometimes if you're putting in symptoms you think your dying instantly M:I'd use the Internet and look at NHS Web sites and stuff.I: so you would look for that badge as a sign of quality information. M:Yeah
Parental Views and Experiences of Healthcare	M:If he came to, well I think, as I said before, I think I would make a GPs appointment. I: What do you think of your family doctor? M: they're brilliant they've always been really good with him. M: I know I've got healthy kids, they are extremely healthy, they don't really get illness or stuff F: I know that when you're growing up, you get a lot of pains down there and you know, my Dad would just put in down to ‘accch you're just maturing’. M:Well yeh, we don't rush you in here for a snotty nose. [It]isn't an accident or an emergency
Practical Ability to get to hospital	I: from here it's a bit of a way to the hospital, what would be your first hospital? M: Probably the Royal. We've got a car so…

**Table 2:** Codebook showing Categories and Themes following data analysis; *categories are descriptions of the common ideas within the interviews with the families, themes are a product of inductive analysis of the data to describe the views and experiences of young men regarding testicular health that would facilitate them to attend hospital in a scrotal emergency*.

Domain of Theme	Themes	Categories
Young Persons’ Lack of Independent Access to Healthcare	Reliance on parents for all aspects of healthcare	Young people protected and disempowerment in health matters Poor knowledge of routes of healthcare access Practical difficulties getting to hospital Parents’ facilitate doctor- adolescent communication Parental legal responsibility for care Distrust of non-familial adults
Young Persons’ Ability to Recognise Concerning Testicular Health Problems	Young persons’ knowledge of testicular function and pathologies	Poor knowledge of testicular function and pathology Poor knowledge about testicular cancer Lack of awareness of consequences of testicular loss Incorrect, myth, misconceptions about testes
	Young Persons’ experience of health education and testicular health specifically	Poor education of testicular function and health in school Knowledge gained through friends, family and community Knowledge gained through celebrity and TV Impact of health campaigns Experiences where learning difficulty interferes with education
	Young people's Views and Experiences of health issues	Previous experiences of self-resolving testicular pain Attitudes to common illness eg cancer, appendicitis Predominance of experience of Injuries in young men Positive personal experiences of health and hospitals
	Health information seeking behaviour	Parents as first point of contact for health advice Use of internet for health questions Poor quality of available information on the internet Need provokers for information seeking;
Young Person's ability to tell their parents about concerns	Ability for the Young Person to discuss male Issues with Parents	Parental embarrassment or projected embarrassment Testicular problems considered ‘health’ not male issues Fear of disruption of family routine Parents as disciplinarians, Parental infallibility Identifying as or evidence of an ‘Open’ family Impact of non-nuclear family
	Psychological aspects of adolescent life affecting ability to discuss testicular health	Anticipatory, projected or contextual embarrassment Confidence versus shyness Consideration of the future in decision making Use of humour Fear of drawing attention to lack of knowledge or experience Discomfort with physical aspects of testes and their body
	Sociological aspects of adolescent life affecting ability to discuss testicular health	Testicles considered to do with sex, social taboo and shame Testicular health considered unimportant, irrelevant and not serious Ephebiphobia Religion Intergenerational change Importance of playing culturally accepted male role in adults
Parents Ability to Take their Adolescent Child to Hospital	Parental Knowledge and Education	Poor parental knowledge of testicular torsion and urgent need to get to hospital Parental predominance of knowledge cancer Misconception testicular problems are adult rather than young person's issues Confusion about normal body changes during puberty
	Information Available for Parents or Guardians Regarding Testicular Torsion	Perceived lack of information Lack of resources for parents/ guardians for health advice Internet, NHS 24 Family doctor as first port for information Perceived predominance of women's health information
	Parental Views and Experiences of Healthcare	Pride in health, stoicism and not burdening the NHS Personal ‘deservability’ assessment: Degree and duration of pain to trigger hospital attendance Parental pride in caring Fathers for male issues (unexperienced)
	Practical Ability to get to hospital	Perceived necessity of car to attend hospital Extended family in help to access healthcare

## 3 Experimental design, materials, and methods

### 3.1 Population and recruitment

Adolescent males 11 to 19 years who had not experienced testicular torsion were recruited. Those who had experienced testicular health issues or had frequent visits to hospital were excluded. Participants were recruited through sports and out of school clubs. Purposive and snowball sampling was used. The Chief Investigator (CI) contacted the clubs and asked to advertise via their email and social media listings and attended practises to meet the young men and their families. Age appropriate information sheets were given to the young people and their families. Contact details were taken for the families and consent and assent forms were signed. The family were given two weeks before being contacted and an interview arranged at home, at the hospital or at the sports club. The young men were asked to choose a chaperone. The adolescents were given a gift voucher as an incentive to take part.

Recruitment occurred in two locations in the UK, and from a broad socioeconomic range. Recruitment continued until data saturation was achieved as defined by standard qualitative methodology [7] whereby the iterative analysis occurs alongside data collection and no further ideas are generated with further participant recruitment.

### 3.2 Probity and ethical issues

Significant considerations were made for care for the young men with age appropriate assent and consent forms, a chaperone at all times, and an offer to withdraw from the study up to four weeks from the interview. Interviews were recorded on an electronic device and transferred to an encrypted hard drive kept on NHS property. Transcriptions were anonymised. The project went through ethical review board (REC number 15/YH/0299, HRA registration 167713), with the research protocol (IRAS 167713) available from the corresponding author.

### 3.3 Data generation and analysis

A qualitative methodology was chosen, [5,8] utilising semi-structured interviews and thematic analysis using a framework approach. The CI performed all interviews with the young person and their chosen chaperones following an interview topic guide developed by the CI from previous knowledge of the clinical phenomena and with the expert families interviewed during pilot interviews (see Fig. 1).

The interviews were audio recorded and transcribed verbatim. Transcription was delayed four weeks post interview allowing families to withdraw from the study if they so wished. Transcription was undertaken by Chief Investigator (CI) Caroline MacDonald for the first 9 interviews and then by a professional transcription service. All were checked against the original recording by the CI. initial interviews and transcriptions were corroborated by supervisor Professor Collins and M. Burton.

Coding was undertaken by the CI, Caroline MacDonald. The first three transcripts were also co-coded by Prof Karen Collins, Academic supervisor, to confirm breadth and depth of coding and to support validity of the data analysis. NVivo 11 © QSR International 2017 was used for coding and data management.

Development of themes and concepts from the coded data took place alongside the collection of further data. Analysis began to move from descriptive to theoretical, allowing recognition of saturation of the material by the coding process, ie when no new insights and interpretations emerge from the data. A self-critical reflexive diary was kept during the interview, coding and analysis process as per recommendations to perform high quality qualitative research [9].

The process of moving from the in-vivo data to themes took place in a systematic step wise fashion as described by Richie and Lewis et al. [4] and demonstrated by Smith and Firth [10].The initial categories were then grouped into themes and a hierarchy began to appear from which an index was developed. All interview transcripts were then re-indexed. In this iterative process the authors tried moving towards explanations and descriptions of the experiences of the young men, which might explain the phenomenon of delay in presentation to hospital with severe testicular pain.

Multiple iterations of indexing were undertaken until all data fitted with the categories intuitively and a framework matrix was constructed. Any gaps or conflicts of data in individual or across cases were inspected with a return to data transcription to ensure no missing data or misunderstood coding. Cases were inspected for consistency and comparisons inspected. Literature review of the emerging themes was undertaken and added to the framework chart to contextualise understanding of the ideas and check validity of the themes in the adolescent social experience.

## Declaration of Competing Interest

The authors declare that they have no known competing financial interests or personal relationships which have, or could be perceived to have, influenced the work reported in this article.
